# Arachidonic acid intake and asthma risk in children and adults: a systematic review of observational studies

**DOI:** 10.1017/jns.2014.9

**Published:** 2014-05-07

**Authors:** Saki Kakutani, Kahori Egawa, Kayo Saito, Toshihide Suzuki, Chika Horikawa, Tomohiro Rogi, Hiroshi Kawashima, Hiroshi Shibata, Satoshi Sasaki

**Affiliations:** 1 Department of Social and Preventive Epidemiology, School of Public Health, The University of Tokyo, Tokyo, Japan; 2 Institute for Health Care Science, Suntory Wellness Limited, Osaka, Japan

**Keywords:** Epidemiology, Asthma, Dietary fatty acids, Free-living populations, ARA, arachidonic acid, cys-LT, cysteinyl leukotriene, STROBE, Strengthening the Reporting of Observational Studies in Epidemiology

## Abstract

The effect of arachidonic acid (ARA) intake on asthma risk is unclear. The objective of the present review was to systematically evaluate available observational studies on the relationship between ARA exposure and asthma risk in children and adults. A PubMed search was conducted on 22 October 2013 and seventy-three publications were checked against predefined criteria for eligibility. To identify additional eligible publications, potentially relevant articles were searched from bibliographies of articles on ARA and asthma. A total of 2924 citations were scrutinised. Finally, fourteen articles were included. A quality assessment was conducted based on the reporting and methodological quality. A meta-analysis was not conducted; therefore, a qualitative assessment is presented. Three high-, two medium- and ten low-quality studies were reviewed. Eleven studies, including two high- and two medium-quality studies, did not find a significant association between ARA exposure and asthma risk. In contrast, one high-quality study indicated a significant trend toward reducing asthma risk in children with decreasing maternal ARA intake (*P*_trend_ = 0·025), and one low-quality study reported a significant trend of increasing asthma risk with higher blood ARA levels (*P*_trend_ = 0·007). In two low-quality studies, asthma patients had significantly lower blood ARA levels than controls (both *P* < 0·05). These studies did not sufficiently demonstrate any relationships between ARA exposure and asthma risk because of the limited number of studies and their methodological limitations. They seem to suggest that ARA exposure is not consistently associated with asthma risk. Nevertheless, further evidence is required to prove or disprove the association.

Asthma is a chronic inflammatory disorder of the airways, usually associated with airway hyper-responsiveness and variable airflow obstruction. Asthma has become more common in both children and adults. It is estimated that as many as 300 million individuals of all ages and all ethnic backgrounds suffer from asthma^(^^1^^)^, and that there may be an additional 100 million individuals with asthma by 2025^(^^2^^)^. The rapid increase in the prevalence of asthma may be explained by changes in environmental factors. The prevention of asthma is one of the major public health issues in the world today.

Many risk factors for chronic respiratory diseases have been proposed based on the modern, urban lifestyle. In particular, changes in eating habits may affect the development of asthma. Several epidemiological studies have reported a beneficial effect of fresh fruit intake on symptoms or lung function in asthma^(^^3^^–^^6^^)^. Some studies have reported a favourable effect of fish consumption during pregnancy on asthma in infants^(^^7^^–^^9^^)^.

Essential fatty acids, namely *n*-3 and *n*-6 fatty acids, are involved in many important biological functions^(^^10^^–^^13^^)^. They play a structural role in cell membranes, influencing their fluidity and membrane enzyme activities. In addition, some are the precursors of prostaglandins and other lipid mediators. Arachidonic acid (ARA) is an *n*-6 essential fatty acid and a major constituent of biomembranes. ARA is also contained in human and animal breast milk and is involved in infant development^(^^14^^–^^16^^)^. Many advisory boards and scientists have recommended the use of infant formula in which both ARA and DHA are contained when breast-feeding is not possible^(^^17^^–^^22^^)^.

ARA is released from membranes by phospholipase A_2_ and converted into various lipid mediators that exert many physiological actions^(^^23^^–^^25^^)^. The cysteinyl leukotrienes (cys-LT) derived from ARA are known to be important pro-inflammatory mediators in the pathogenesis of asthma^(^^26^^,^^27^^)^. Current guidelines recommend leukotriene receptor antagonists as a second-choice treatment or an add-on therapy to reduce the dose of inhaled corticosteroids^(^^28^^–^^30^^)^, suggesting that high ARA exposure may cause asthma through the leukotriene pathway because it increases ARA content in cell membranes. The hypothesis that EPA and DHA, *n*-3 essential fatty acids, reduce ARA content in cell membranes and inhibit ARA metabolism has been used as an explanation of the beneficial effects of fish intake or supplementation with fish oil on asthma in many experiments^(^^7^^–^^9,^^31^^)^. However, some observational studies failed to show that ARA exposure was positively correlated with asthma risk^(^^32^^–^^34^^)^. ARA is one of the major PUFA, particularly in early life, and this inconsistency is not negligible.

No systematic review or meta-analysis has been conducted to evaluate the long-term effects of ARA intake and blood or non-blood tissue ARA composition on asthma risk in free-living populations. The objective of the present study was to systematically evaluate available observational studies on the relationship between ARA exposure and asthma risk.

## Methods

### Search strategy

**Fig. 1.**
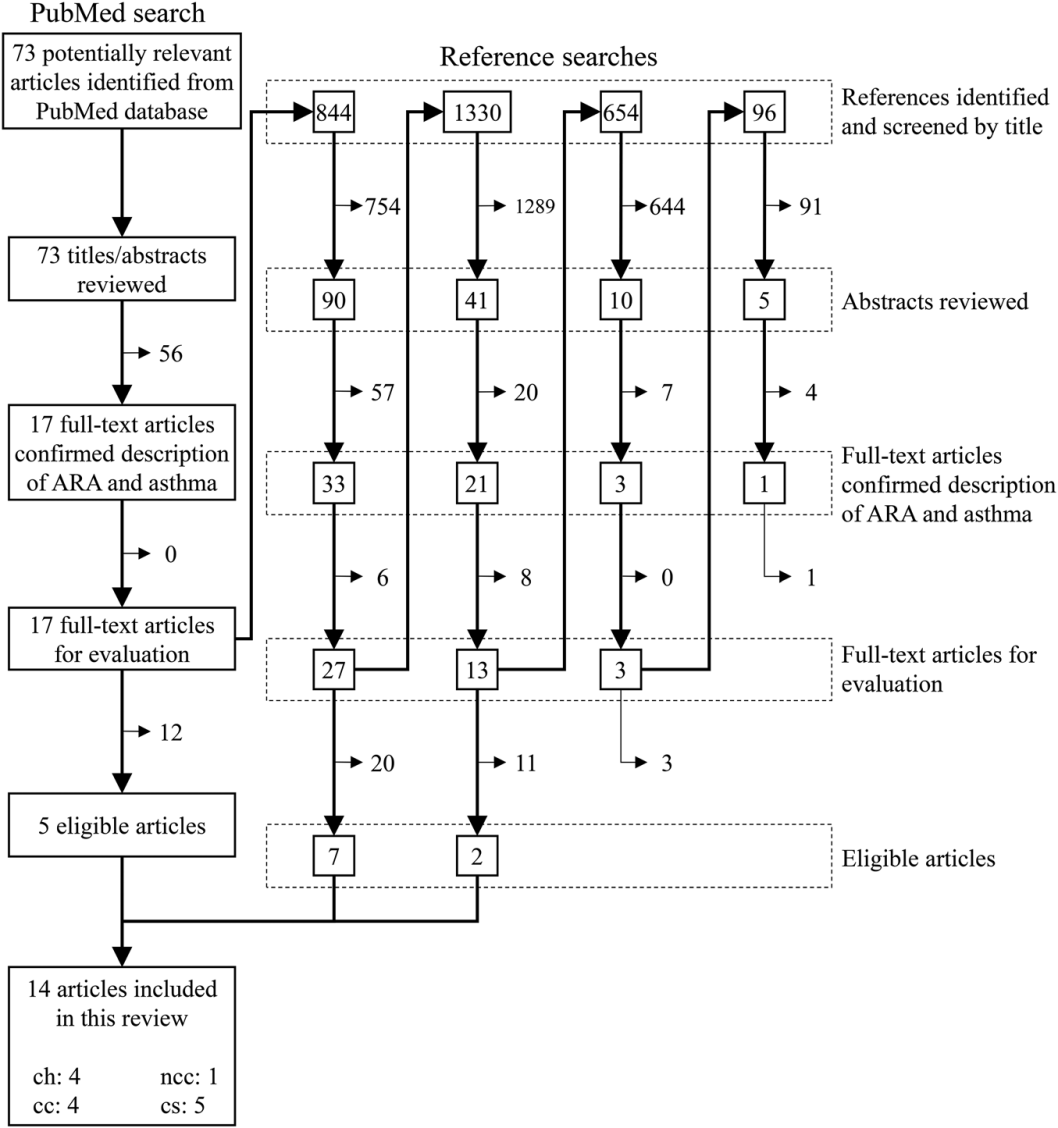
Flow diagram for the literature search and study selection. ARA, arachidonic acid; ch, cohort study; ncc, nested case–control study; cc, case–control study; cs, cross-sectional study.

The PubMed database (http://www.ncbi.nlm.nih.gov/pubmed/) was searched for observational studies on the relationship between dietary or blood ARA levels and asthma risk that were listed in PubMed up to 17 May 2010. To identify target articles effectively, the strategy for the PubMed search was as follows: keywords for outcome and study types were adopted as commonly used terms representing asthma and study design, whereas terms for exposure were selected from specific words that stand for ‘arachidonic acid’ (see online Supplementary Table S1). The PubMed search was updated on 22 October 2013, yielding seventy-three potentially relevant articles (Fig. 1).

### Study selection

Inclusion criteria were English-language articles that reported original data on the relationship between ARA exposure and asthma risk in free-living populations. ARA exposure was assessed as dietary intake, blood levels or non-blood tissue levels for participants' own risk, and as mothers' dietary intake, mothers' blood levels or mothers' non-blood tissue levels for their children's risk. Eligible study designs were cohort, case–control or cross-sectional studies. Articles published after 1966 were searched considering PubMed database coverage.

The study selection process is presented in Fig. 1. Articles that were excluded were those whose titles or abstracts indicated clearly that they: (1) were not human studies; (2) were limited to special populations such as individuals with unusual eating habits; (3) involved assessment only after intervention; or (4) were not about asthma and fatty acids (not fat). Titles and abstracts of the publications identified from the PubMed database were checked and reviewed against the predefined criteria. Next, it was confirmed that the articles had a description of ARA and asthma in their full text. To identify additional eligible publications, potentially relevant articles were searched from bibliographies of full-text articles that included descriptions of both ARA and asthma. Our previous review suggested that searches from bibliographies of articles including ARA were more efficient when enough articles were identified from the PubMed database^(^^35^^)^. They were screened using the same criteria as for the PubMed search. This reference search procedure was continued until no new potentially relevant articles could be identified from bibliographies.

The titles and abstracts of the seventy-three publications identified from the PubMed database were checked and reviewed against the predefined criteria. Seventeen publications were confirmed, and five of these original articles in English from the PubMed search were finally included in the present review. A total of 2924 citations were scrutinised, and nine articles were obtained. Thus, fourteen eligible articles were finally included in the review. These database and reference searches were performed by one evaluator (K. E. or S. K.) and then checked by another (S. K. or T. S.).

### Quality assessment and data extraction

Quality assessment was conducted based on the reporting quality and the methodological quality of each study. The reporting quality shows whether the necessary information for observational studies is well reported; it is the number of items from the Strengthening the Reporting of Observational Studies in Epidemiology (STROBE) checklist^(^^36^^)^. The reporting quality of included observational studies was assessed by one reviewer (K. E. or S. K.) and then confirmed by other reviewers (S. K. and K. S., or C. H.). The methodological quality, the level of the suitability of the methods used in a study, was assessed by two reviewers (S. K. and K. S., or S. K. and C. H.) qualitatively based on the following methodological aspects reported in the article: subject selection, ARA exposure assessment, diagnosis or recruitment procedure of participants, methods for controlling confounders, and statistical analysis. Study quality was finally defined as below: studies with reporting quality scores under 13 or with insufficient temporal information between exposure and outcome were considered low quality; other studies were qualitatively divided into high/medium/low quality according to their methodological quality.

For each eligible article, the following information was tabulated: authors and year of publication, study settings and design, subject characteristics (such as age, sex and number), matching strategy (if applicable), ARA exposure assessment used (as well as information about validity or precision), outcome assessment, adjusted confounders, reporting quality score from the STROBE checklist, and main findings from the fully adjusted model. Case–control studies were classified into two groups based on whether they reported temporal information between exposure and outcome assessment: a ‘case–control study (temporal relationship between exposure and outcome is demonstrated)’ was defined as an article in which ARA exposure preceded the occurrence of asthma, whereas a ‘case–control study (temporal relationship between exposure and outcome is unclear)’ did not describe sufficient temporal information between exposure and outcome assessment.

A meta-analysis was not conducted because of the heterogeneity among studies, particularly in the subject characteristics and exposure/outcome assessments, and the insufficient number of studies of high quality suitable for a meta-analysis. Therefore, a qualitative assessment of ARA exposure and asthma risk is presented in the review.

## Results

A total of fourteen eligible articles were selected from potentially relevant papers and included in the present systematic review (Fig. 1); their major characteristics are shown in Table 1^(^^32^^–^^34,^^37^^–^^47^^)^. One article reported both dietary ARA intake and erythrocyte membrane ARA levels, and it was therefore treated as two individual studies^(^^40^^)^.

**Table 1.** Summary of observational studies on the association between arachidonic acid (ARA) exposure and asthma risk

References	Study	Subjects	Exposure assessment	Asthma assessment (diagnosis)	Adjustment for potential confounders	Assessment of reporting quality*	Main findings		
							Intergroup comparison		*P* or *P*_trend_
Study design: cohort study									
Exposure assessment: maternal dietary intake									
Lumia *et al.* (2011)^(^^37^^)^	DIPP Nutrition Study, Finland, 1997–2009, cohort design (5 years follow-up)	2679 mother–child pairs	Self-administered semi-quantitative FFQ, 181 items, validated against 12 × 5 d DR, diet of mothers during 8th month of pregnancy	Parent-reported questionnaire based on ISAAC questionnaire with parental report of child's age at physician diagnosis at 5 years	Child's sex, region of birth, duration of gestation, maternal age, maternal education level, maternal smoking status, number of previous deliveries, parental history of asthma and/or allergic rhinitis, child's birth weight, delivery mode, pets at home, farming, contact with cow stable, breast-feeding duration	21	Dietary ARA intake, g/d, tertile, range	HR	*P*_trend_
							T1: <0·06	0·52 (95 % CI 0·32, 0·84)	0·025
							T2: 0·06–0·11	1·00	
							T3: >0·11	0·77 (95 % CI 0·51, 1·17)	
Exposure assessment: maternal blood ARA level									
Notenboom *et al.* (2011)^(^^32^^)^	KOALA Birth Cohort Study, Netherlands, 2002–, cohort design (6–7 years follow-up)	951 mother–child pairs (maternal age at 6–7 years follow-up 32·7 years)	Plasma phospholipids, GC analysis, precision not indicated, plasma at 34–36 weeks of pregnancy	Parent-reported questionnaire based on ISAAC questionnaire at 6–7 years	Recruitment group, maternal age, maternal ethnicity, maternal education level, maternal smoking status, parental history of atopy and/or asthma, duration of gestation, season of birth, child's sex, child's birth weight, delivery mode, exposure to environmental tobacco, presence of older siblings and sibling atopy, breast-feeding, child daycare, pets at home	22	Plasma ARA composition, wt%, quintile, range	OR	*P*_trend_
							Q1: ≤6·46	1·00	0·83
							Q2: 6·47–7·15	1·69 (95 % CI 0·70, 4·10)	
							Q3: 7·16–7·85	1·29 (95 % CI 0·52, 3·20)	
							Q4: 7·86–8·60	0·82 (95 % CI 0·31, 2·15)	
							Q5: ≥8·61	1·70 (95 % CI 0·67, 4·33)	
Exposure assessment: maternal non-blood tissue ARA level									
Lowe *et al.* (2008)^(^^38^^)^	MACS, Australia, 1990–2001, cohort design (7 years follow-up)	224 mother–child pairs with history of allergic disease of a first-degree family of child, 194 colostrum samples and 118 expressed breast milk samples	Colostrum fatty acids and expressed breast milk fatty acids, GC analysis, precision not indicated, colostrum at 2–4 d after delivery and expressed breast milk at 3 months	Parent's report of physician diagnosis and wheezing event during telephone interview at 6 and 7 years	None (adjusting for child's sex, parental education level, and use of gas heating at home did not alter the association)	15	Colostrum ARA composition, wt%	OR per 1 sd increase	*P*
								0·87 (95 % CI 0·63, 1·21)	0·413
							Expressed breast milk ARA composition, wt%	OR per 1 sd increase	*P*
								1·06 (95 % CI 0·69, 1·64)	0·783
Wijga *et al.* (2006)^(^^39^^)^	PIAMA Study, Netherlands, 1996–2001, cohort design (4 years follow-up)	158 mother with allergy–child pairs (maternal age 31·2 years)	Breast milk fatty acids, GC analysis, precision not indicated, breast milk at 3 months visit	Parent-reported questionnaire based on ISAAC questionnaire with parental report of physician diagnosis at 4 years	None (child's sex, number of older siblings, maternal age, maternal smoking status, and maternal BMI hardly did not alter the association when they were entered one at a time into the model)	19	Breast milk ARA composition, wt%	Prevalence of asthma, %	*P*
							Below median	16·1	NS
							Above median	8·5	
							Breast milk ARA composition, wt%	OR per an interquartile increase	*P*
								0·74 (95 % CI 0·37, 1·47)	NS
Study design: nested case–control study									
Exposure assessment: dietary intake									
Nagel & Linseisen (2005)^(^^33^^)^	EPIC-Heidelberg cohort, Germany, 1994–2000 (2·1 years follow-up)	105 newly diagnosed adult asthma patients, 420 controls without prevalent asthma or other atopic diseases, aged 35–65 years in women and 40–65 years in men at recruiting, one case matched with four controls by sex, age	Self-administered semi-quantitative FFQ, 158 items, validated against 12 × 24HDR	Self-reported physician diagnosis	Age, fat energy intake, non-fat energy intake, BMI, smoking status, sex, educational level	22	Dietary ARA intake, mg/d,tertile	OR	*P*_trend_
							T1	1·00	0·838
							T2	0·70 (95 % CI 0·39, 1·23)	
							T3	0·85 (95 % CI 0·44, 1·68)	
Study design: case–control study (temporal relationship between exposure and outcome is unclear)									
Exposure assessment: dietary intake									
Broadfield *et al.* (2004)^(^^40^^)^	Survey, UK, 2000–2001, case–control design	Adults without hypertension, CVD or other metabolic or inflammatory disorders, eighty-nine asthma patients without LT antagonists or oral corticosteroids (mean age 42·8 years), eighty-nine controls, one case matched with one control by primary care register, sex, age	Self-administered semi-quantitative FFQ, 129 items, validated against 7 d weighed dietary record	Physician diagnosis	Season of data collection, BMI, total energy intake	20	Dietary ARA intake, mg/d, mean	Dietary ARA intake, mg/d, mean	*P*
							Case	Control	
							0·13 (sd 0·08)	0·11 (sd 0·05)	NS
							Dietary ARA intake, mg/d	OR for 75th *v.* 25th percentile	*P* or *P*_trend_
								1·53 (95 % CI 0·92, 2·55)	Not shown
Exposure assessment: blood ARA level									
Broadfield *et al.* (2004)^(^^40^^)^	Survey, UK, 2000–2001, case–control design	Adults without hypertension, CVD or other metabolic or inflammatory disorders, eighty-nine asthma patients without LT antagonists or oral corticosteroids (mean age 42·8 years), eighty-nine controls, one case matched with one control by primary care register, sex, age	Erythrocyte membrane fatty acids (overnight fasting venous blood), GC-MS analysis, precision not indicated	Physician diagnosis	Season of data collection, BMI, total energy intake	20	ARA composition, %, mean	ARA composition, %, mean	*P*
							Case	Control	
							15·4 (sd 3·4)	16·3 (sd 3·9)	NS
							ARA composition, %	OR for 75th *v.* 25th percentile	*P* or *P*_trend_
								0·76 (95 % CI 0·45, 1·26)	Not shown
Picado *et al.* (1999)^(^^41^^)^	Survey, Spain, case–control design	118 adult asthma patients aged 16–72 years, 121 controls aged 17–74 years, matching not indicated, equivalent age, weight, height, BMI	Serum fatty acids, GC analysis, precision not indicated	Physician diagnosis, characterised based on method in GINA	Age, sex, corticosteroid therapy	13	ARA composition, %, mean	ARA composition, %, mean	*P*
							Case	Control	
							7·2 (sem 1·8)	6·9 (sem 1·6)	NS
Leichsenring *et al.* (1995)^(^^42^^)^	Survey, Germany	Seventeen children atopic asthma patients (mean age 9 years), ten controls with no atopic diseases, matched by age	Plasma phospholipids and plasma CE, GC analysis, precision not indicated	Physician diagnosis, graded according to classification by von der Hardt	None	8	Phospholipids ARA composition, wt%, mean	Phospholipids ARA composition, wt%, mean	*P*
							Case	Control	
							8·62 (sd 1·55)	9·65 (sd 1·56)	NS
							CE ARA composition, wt%, mean	CE ARA composition, wt%, mean	*P*
							Case	Control	
							5·46 (sd 0·99)	6·76 (sd 1·34)	<0·05
Griese *et al.* (1990)^(^^43^^)^	Survey, Germany	Eleven children allergic asthma patients without atopic dermatitis aged 1·5–16·5 years, ten controls aged 1–16 years, matched by age	Plasma phospholipids and MNC phospholipids (>5 h fasting blood), GC analysis, precision not indicated	Physician diagnosis	None	7	Plasma phospholipids ARA composition, %, mean	Plasma phospholipids ARA composition, %, mean	*P*
							Case	Control	
							9·77 (sd 1·46)	8·96 (sd 1·86)	NS
							MNC phospholipids ARA composition, %, mean	MNC phospholipids ARA composition, %, mean	*P*
							Case	Control	
							18·88 (sd 4·83)	19·24 (sd 3·60)	NS
Study design: cross-sectional study									
Exposure assessment: dietary intake									
Miyake *et al.* (2008)^(^^34^^)^	RYUCHS, Japan, 2004–2005, cross-sectional design	25033 schoolchildren of fifty-two public elementary schools and twenty-five junior high schools aged 6–15 years	Self-administered BDHQ for children, fifty-one items, developed based on DHQ validated against 3 d DR	Self- or parent-reported questionnaire based on ISAAC phase I questionnaire	Age, sex, resident area, number of siblings, family smoking status, BMI, parental history of allergic diseases, parental educational level, total energy intake	21	Dietary ARA intake, g/d, quintile, median	OR	*P*_trend_
							Q1: 0·06	1·00	0·74
							Q2: 0·09	0·87 (95 % CI 0·75, 1·01)	
							Q3: 0·12	1·08 (95 % CI 0·94, 1·25)	
							Q4: 0·15	1·00 (95 % CI 0·86, 1·16)	
							Q5: 0·18	0·96 (95 % CI 0·83, 1·12)	
Exposure assessment: blood ARA level									
De Castro *et al.* (2007)^(^^44^^)^	Survey, Spain	Fifteen adult asthma patients with no smoking history, fifteen controls with >19 pack-years smoking and currently smoking, equivalent age, weight, blood lipids, blood pressure, BMI	Erythrocyte membrane fatty acids and platelets membrane fatty acids, GC-MS analysis, precision not indicated	Self-reported questionnaire with physician's diagnosis	None	11	Erythrocyte ARA composition, %, mean	Erythrocyte ARA composition, %, mean	*P*
							Case	Control	
							6·46 (sd 3·48)	10·57 (sd 2·79)	<0·001
							Platelets ARA composition, %, mean	Platelets ARA composition, %, mean	*P*
							Case	Control	
							9·87 (sd 3·10)	16·08 (sd 3·99)	<0·004
Bolte *et al.* (2006)^(^^45^^)^	ISAAC phase II, Germany, cross-sectional design	526 children (nested case–control study population in ISAAC phase II), aged 8–11 years	Serum CE, HPLC analysis, precision not indicated	Parent's report of physician diagnosis	Sex, age, parental education level, parental asthma	21	ARA composition, %, quartile	Current asthma OR	*P*_trend_
							Q1	1·00	0·007
							Q2	3·34 (95 % CI 1·30, 8·58)	
							Q3	2·11 (95 % CI 0·79, 5·64)	
							Q4	4·54 (95 % CI 1·77, 11·62)	
Woods *et al.* (2004)^(^^46^^)^	Survey, Australia	Randomly selected adult subjects in Melbourne aged 20–44 years, 986 for current asthma, 1049 for asthma and physician-diagnosed asthma	Plasma phospholipids, GC analysis, precision not indicated	Interviewer-administered questionnaire	Age, sex, BMI, smoking status, family history of asthma, region of birth, total energy intake	20	Plasma phospholipids ARA composition, %	OR per 1 % increase	*P* or *P*_trend_
								Current asthma	
								0·97 (95 % CI 0·88, 1·06)	Not shown
								Asthma	
								1·05 (95 % CI 0·98, 1·14)	Not shown
								Diagnosed asthma	
								0·94 (95 % CI 0·87, 1·02)	Not shown
Exposure assessment: non-blood tissue ARA level									
Wijga *et al.* (2003)^(^^47^^)^	PIAMA Study, Netherlands, 1996–1997	168 allergic mothers including forty-seven mothers with history of asthma and 107 non-allergic mothers	Breast milk fatty acids, GC analysis, precision not indicated, breast milk at 2–35 weeks	Self-reported questionnaire	None	9	Breast milk ARA composition, wt%, mean	Breast milk ARA composition, wt%, mean	*P*
							Non-allergic	Asthma	0·144
							0·39 (sd 0·10)	0·36 (sd 0·10)	

DIPP, Diabetes Prediction and Prevention; DR, diet record; ISAAC, International Study of Asthma and Allergies in Childhood; HR, hazard regression; KOALA, Kind, Ouders en gezondheid: Aandacht voor Leefstijl en Aanleg (Child, parents and health: Lifestyle and genetic constitution); MACS, Melbourne Atopy Cohort Study; PIAMA, Prevention and Incidence of Asthma and Mite Allergy; EPIC, European Prospective Investigation into Cancer and Nutrition; 24HDR, 24 h dietary recall; LT, leukotriene; GINA, Global Initiative for Asthma; CE, cholesteryl ester; MNC, mononuclear cell; RYUCHS, Ryukyus Child Health Study; BDHQ, Brief-Type Self-Administered Diet History Questionnaire; DHQ, diet history questionnaire; STROBE, Strengthening the Reporting of Observational Studies in Epidemiology.

*Result of the critical evaluation carried out using the checklist of the STROBE statement.

The study quality of two cohort studies on maternal ARA intake or maternal blood ARA levels and one nested case–control study was considered to be high, because ARA exposure had clearly preceded the onset of asthma in these studies, and their relationship was carefully analysed^(^^32^^,^^33,^^37^^)^. The remaining two cohort studies in which ARA levels of breast milk were measured were regarded as having medium quality; maternal ARA exposure, which transferred to breast milk, clearly preceded the asthma onset of children, but confounding factors in their relationship were not sufficiently considered in these studies^(^^38^^,^^39^^)^. The quality of all studies using a case–control and a cross-sectional design was low^(^^34^^,^^40–^^47^^)^. Their reporting quality was very low, and/or temporal information between exposure and outcome was insufficient. As a result, three high-, two medium- and ten low-quality studies were reviewed.

The risk of asthma in children was evaluated in eight studies, and that in adults was examined in seven studies. The studies are not discussed separately because the results do not seem to be clearly different between children and adults. Dietary intake was estimated using self-administered FFQ or a brief self-administered diet history questionnaire, both of which were validated against multiple-day dietary records or 24 h dietary recalls. By contrast, no article in which ARA levels in blood or non-blood tissue were measured mentioned the masking procedure for participants' information and the precision of analysis. Most studies considered one or more potential confounders, such as age, BMI and parental asthma, though the extent varied greatly.

Dietary ARA intake was estimated in one nested case–control study, one case–control study and one cross-sectional study. The nested case–control study was considered to be of high quality. The quality of the remaining two studies was low. These three studies did not show a significant association between ARA exposure and asthma risk. The mean or median intake of dietary ARA was within a narrow range from 110 to 150 mg/d.

Maternal dietary intake of ARA was estimated in one cohort study which indicated a significant trend toward reducing asthma risk in children with decreasing maternal ARA intake (*P*_trend_ = 0·025)^(^^38^^)^. The quality was considered to be high.

In the four case–control studies and the three cross-sectional studies, exposure was reported as blood ARA levels. All of these studies were considered to have low study quality. Bolte *et al.*^(^^45^^)^ showed a significant trend of increasing asthma risk with increasing serum cholesteryl ester ARA levels (*P*_trend_ = 0·007)^(^^45^^)^. Leichsenring *et al.*^(^^42^^)^ and de Castro *et al.*^(^^44^^)^ reported a significant decrease in blood ARA levels in asthmatic subjects (Leichsenring *et al.*^(^^42^^)^: *P* < 0·05 for plasma cholesteryl esters; de Castro *et al.*^(^^44^^)^: *P* < 0·001 for erythrocyte membranes and *P* < 0·004 for platelet membranes). The other four studies showed no significant association between ARA exposure and asthma risk.

Maternal blood ARA levels during pregnancy were measured as ARA exposure in one cohort study. No significant trend toward increased asthma risk in their children was apparent. The quality of this study was considered to be high.

Two cohort studies and one cross-sectional study reported ARA levels in breast milk. These levels were considered representative of maternal non-blood tissue ARA levels reflecting maternal ARA intake, because the amounts of breast milk that each infant received were not reported. Two cohort studies did not show significant trends of increasing asthma risk in children with increasing breast milk ARA levels. These studies were regarded as being of medium quality. No significant difference in ARA levels in breast milk between non-allergic mothers and asthmatic mothers was seen in the cross-sectional study. The quality of the cross-sectional study was low.

## Discussion

In the present review, observational studies investigating the association between ARA and asthma in free-living populations were systemically reviewed. Fourteen eligible articles were obtained from the search strategy, nine of which were identified from reference searches (Fig. 1). Thus, reference searches served an important role in ensuring a comprehensive literature search.

Among the nine eligible articles from the reference searches, eight were not identified by the PubMed search formula due to keywords related to ‘exposure’, and one was not identified due to keywords related to ‘study types’. The reason that more than half of the eligible articles could not be identified in PubMed searches is considered to be due to the particularity of the ‘exposure’ keywords. Authors often describe only two or three interesting fatty acids in titles or abstracts, whereas many other fatty acids are simultaneously evaluated and described in texts or tables. This is unavoidable, because there are more than ten meaningful fatty acids in foods or the human body. This reporting characteristic made it difficult to effectively search for observational studies with a focus on individual fatty acids such as ARA, which is similar to our previous review that evaluated the observational studies on the relationship between ARA exposure and cancer risk^(^^35^^)^. For example, six studies that were not identified due to ‘exposure’ could be included in the PubMed search by the addition of the search term ‘fatty’, but the initial number of articles from PubMed more than doubled. In the case of ‘study type’ terms, the unidentified literature was published in 1995 and was the oldest of the eligible articles. Because the STROBE statement, which recommends that authors should indicate the study design in the title or abstract, was developed in 2007, the necessity of defining study designs would have not been widely recognised when the article was published.

There were fourteen eligible articles. This was considered insufficient to draw conclusions about the relationship between ARA exposure and asthma risk because of the limited number of studies of high quality. On the whole, a strong positive association and a clear dose–response relationship between increased or decreased asthma risk and ARA exposure were not observed, although the results were obtained under widely varying experimental conditions among studies, such as subjects' background, ARA evaluation, and method of asthma diagnosis. This might suggest that ARA exposure is not associated with asthma risk.

In two studies asthma risk increased significantly with increasing ARA exposure, where the risks in children were evaluated^(^^37^^,^^45^^)^. However, most of the other studies in children, as in adults, did not show a significant relationship between ARA exposure and asthma or a difference in ARA levels in asthmatic subjects. It should be also considered that ARA is required for infant growth, development and health^(^^14^^–^^16^^)^.

A clear temporal sequence of exposure before outcome is one of the important factors to establish a causal relationship between the risk factor and the target event. This was reported in only five of fourteen eligible articles. Five studies used a case–control design, but the temporal relationship between ARA estimation and asthma diagnosis was not expressed clearly. The remaining five studies used a cross-sectional design. The reliability of these ten studies was considered limited. The proportion of studies with an unclear temporal sequence was high, which may be due to the characteristic of asthma that it is difficult to clearly delineate when asthma begins.

The biological plausibility of the relationship between ARA exposure and asthma risk still cannot be fully explained. The cys-LT derived from ARA produce effects that are characteristic of asthma, such as potent bronchoconstriction, increased endothelial membrane permeability leading to airway oedema, and enhanced secretion of thick, viscous mucus, and their receptor antagonists are used clinically to treat asthma^(^^26^^–^^30^^)^. Many observational studies, however, have not shown any association between ARA exposure and asthma risk^(^^32^^–^^34,^^38^^–^^41,^^43^^,^^46,^^47^^)^. These contrasting findings may be explained in part by the following three reasons. First, blood or lung tissue ARA levels may not always represent dietary intake. Blood levels of PUFA are influenced not only by diet, but also by genetic variants of fatty acid conversion enzymes^(^^48^^,^^49^^)^. Kobayashi *et al.*^(^^50^^)^ and Garland *et al.*^(^^51^^)^ reported that correlations between dietary estimates and the ARA contents of adipose tissue or serum phospholipids were low. On the other hand, Rett *et al.*^(^^52^^)^ reported that ARA levels in plasma/serum phospholipids are increased by ARA supplementation in adult individuals consuming Western-type diets. Second, the increment of blood or non-blood tissue ARA levels may not be connected with the levels of ARA metabolites. Kelley *et al.*^(^^53^^)^ reported that ARA supplementation increases the production of leukotriene B_4_ in human monocytes *ex vivo*; however, it has remained unclear whether an increase in dietary ARA is directly associated with cys-LT synthesis in humans. Our previous study indicated that supplementation with 240 or 720 mg ARA per d did not significantly change plasma prostanoids, which are ARA metabolites produced through pathways other than leukotrienes, although plasma ARA levels increased^(^^54^^)^. Nielsen *et al.*^(^^55^^)^ reported that adipose tissue ARA was not correlated with either cys-LT formation in plaque or total body cys-LT formation in a cross-sectional study of subjects undergoing femoral thromboendarterectomy. Third, ARA metabolites other than cys-LT may decrease asthma risk. Lipoxins are trihydroxytetraene-containing eicosanoids that are generated during asthma. It has been demonstrated that lipoxin A_4_ derived from ARA blocks asthmatic responses in human subjects and experimental model systems^(^^56^^,^^57^^)^.

The present systematic review has four limitations. First, the study selection could not be conducted independently by two or more reviewers. The inclusion/exclusion criteria were clear, and there were few differences that depended on who was in charge, but this may have introduced a potential selection bias. Second, the database search using only the PubMed database and restricted to English-language publications might not completely eliminate article selection bias. Furthermore, articles that investigated ARA levels of non-blood tissues as an exposure assessment could not be identified comprehensively, because the search terms for them were not set before the PubMed search. Third, the search term ‘fatty’ or ‘fatty acid’ was not used in the PubMed search. However, we believe that the literature search was nearly complete because of the comprehensive reference searches. Fourth, quality assessment of observational studies is difficult because of the heterogeneity of study designs and methods. The reporting quality was quantitatively expressible using the STROBE checklist, whereas the methodological quality could not be quantified and was qualitatively estimated by two independent reviewers. This may have affected the results and conclusions of the present review.

Thus, there are insufficient studies to draw any firm conclusions about the relationship between ARA and asthma risk. Further evidence from well-designed observational studies, in particular from those with a clear time sequence of exposure and outcome, is required.

In conclusion, articles that investigated the association between dietary ARA intake or its biomarkers and the risk of asthma were systematically identified, and only a limited number of observational studies were found. Furthermore, most studies had one or more critical limitations; especially critical was the insufficiency of the temporal information between exposure and outcome. These studies did not sufficiently demonstrate any relationships between ARA exposure and asthma risk. They seem to suggest that ARA exposure is not consistently associated with increased asthma risk. Nevertheless, further evidence from well-designed observational studies is required to prove or disprove the association between ARA exposure and asthma risk.

## Supplementary Material

Supplementary Material Supplementary information supplied by authors.

## Supplementary material

To view supplementary material for this article, please visit http://dx.doi.org/10.1017/jns.2014.9
