# Supplementary material for: Arachidonic acid intake and asthma risk in children and adults: a systematic review of observational studies
Source: J Nutr Sci. 2014 May 7;3:e12. doi: 10.1017/jns.2014.9 (PMC4153330; doi:10.1017/jns.2014.9)
Supplement: Supplementary Material — Supplementary information supplied by authors. [file S2048679014000093sup001.doc]

**Supplementary Table S1. PubMed search terms and strategies**

A. Search terms for exposure, outcome, and study types

| Number | Items | Terms |
| --- | --- | --- |
| Exposure | | |
| #1 | Intake | Humans[mesh] AND (arachidonic OR arachidonate OR arachidonates OR "20:4" OR "C20:4" OR eicosatetraenoic) AND ((dietary OR diet OR diets) OR (intake OR intakes OR consumption)) |
| #2 | Biomarker | Humans[mesh] AND (arachidonic OR arachidonate OR arachidonates OR “20:4” OR “C20:4” OR eicosatetraenoic) AND (blood OR serum OR plasma OR erythrocyte OR erythrocites OR “red cell” OR “red cells”) AND ((composition OR compositions OR profile OR profiles OR ratio OR ratios OR status OR statuses) OR (concentration OR concentrations OR level OR levels)) |
| Outcome | | |
| #3 | Asthma | Asthma[mesh] OR asthma*[tiab] |
| Study types | | |
| #4 | Study design | (Epidemiology OR epidemiologic OR epidemiological) OR (prospective OR cohort) OR ("case control" OR (case AND control) OR "control subjects" OR "control group") OR "cross sectional" |

B. PubMed search strategy

| Term combination |
| --- |
| (#1 OR #2) AND #3 AND #4 |
